# IUC23262-80 Comparison of clinical outcome and toxicity of prostate cancer patients undergoing radical prostate radiotherapy using spacer gel versus prostate cancer patients on radiotherapy without spacer gel

**DOI:** 10.1093/oncolo/oyaf248.034

**Published:** 2025-08-29

**Authors:** N Korisatan, R Venkitaraman, M Faisal

**Affiliations:** Ipswich Hospital, United Kingdom; Ipswich Hospital, United Kingdom; Ipswich Hospital, United Kingdom

## Abstract

**Background:**

Spacer gels are used in prostate radiotherapy to reduce rectal toxicity by increasing the space between the prostate and rectum. This audit aimed to Assess the clinical outcomes and toxicity levels in prostate cancer patients receiving radical radiotherapy with spacer gel versus without the use of spacer gel. Evaluate rectal and bladder dosimetry, genitourinary (GU) and gastrointestinal (GI) toxicity, and PSA levels. Align findings with NICE guidance promoting patient safety, quality care, and innovation.

**Methods:**

A retrospective audit of 29 prostate cancer patients treated between April 5, 2023 and August 7, 2024. Spacer Group: 12 patients received spacer gel during radiotherapy. Non-Spacer Group: 17 patients received standard radiotherapy. Dose: 60 Gy in 20 fractions. Data collected Dose-volume histogram (DVH), GI/GU toxicity (CTCAE scale), and PSA at 52 weeks. DVH parameters: Mean/max rectal dose, % rectal volume receiving 80%, 90%, 95%, 100% of prescribed dose; bladder dose volumes (V40Gy, V48Gy, V60Gy). Toxicity monitored at end of treatment, and at 6, 24, and 52 weeks post-treatment.

**Results:**

Rectal Dose: Spacer gel reduced high-dose rectal exposure (V95%). Bladder Dose: The Spacer group showed increased bladder dose, requiring clinical correlation. GU Toxicity: Spacer Group: 11/12 had Grade 1 at 6 weeks; all improved to Grade 0-1 by 6 months. Non-Spacer Group: 17/17 had Grade 1; no worsening at 6 months. GI Toxicity: Spacer Group: 3/12 had Grade 1 at 6 weeks; 0% had toxicity at 6 months. Non-Spacer Group: 7/17 had Grade 1-2 at 6 weeks; 2 had persistent Grade 1 at 6 months. PSA Outcome: All patients in spacer group achieved PSA < 2 ng/mL at 52 weeks.

**Conclusions:**

Spacer gel effectively reduces high-dose rectal exposure and late GI toxicity in prostate radiotherapy without compromising treatment efficacy. No Grade 2+ GI toxicity observed in the spacer group. Supports future use in high-risk prostate cancer patients.

